# Wear resistance of an additively manufactured high-carbon martensitic stainless steel

**DOI:** 10.1038/s41598-022-15621-9

**Published:** 2022-07-22

**Authors:** Eleftherios Iakovakis, Egemen Avcu, Matthew J. Roy, Mark Gee, Allan Matthews

**Affiliations:** 1 Department of Mechanical, Aerospace and Civil Engineering, The University of Manchester, Manchester, M13 9PL UK; 2 Department of Engineering, National Physical Laboratory, Teddington, TW11 0LW UK; 3 Department of Mechanical Engineering, Kocaeli University, Kocaeli, 41001 Turkey; 4 Department of Materials, The University of Manchester, Manchester, M13 9PL UK; 5 Department of Materials, Henry Royce Institute, The University of Manchester, Manchester, M13 9PL UK

**Keywords:** Materials science, Engineering

## Abstract

The dry sliding wear behaviour of a high carbon martensitic stainless steel (HCMSS) consisting of ~ 22.5 vol% of chromium (Cr)- and vanadium (V)-rich carbides processed by electron beam melting (EBM) has been captured. The microstructure consisted of martensite and retained austenite phases with a homogeneous distribution of sub-micron-sized V-rich and micron-sized Cr-rich carbides, leading to relatively high hardness. The CoF decreased ~ 14.1% with increasing load in the steady-state, due to the material transferred from the wear track over the counterbody. The wear rate of the HCMSS compared to martensitic tool steel processed in the same manner, and it was nearly identical under low applied load. The dominant wear mechanism was removal of the steel matrix through abrasion, followed by the oxidation of the wear track, while three-body abrasive wear occurred with increasing load. A plastically deformed zone beneath the wear track was revealed through cross-sectional hardness mapping. Specific phenomena occurred with increasingly aggressive wear conditions were described with carbide cracking, pull-out of V-rich carbides and matrix cracking. This study revealed the wear performance of the additively manufactured HCMSS, which could pave the way for producing components for wear-related applications ranging from shafts to plastic injection moulds via EBM.

## Introduction

Stainless steels (SS) are a versatile steel family widely used in a wide range of aerospace, automotive, food processing and many other engineering applications due to their high corrosion resistance and suitable mechanical properties^1–3^. Their high corrosion resistance is attributed to the high chromium content (above 11.5% wt) in SS, facilitating the formation of a chromium-rich oxide film on the surface^1^. However, most SS grades have a low carbon content and therefore limited hardness and wear resistance, leading to a shorter service life in wear-related applications such as aeronautical landing components^4^. They usually possess low hardness (ranging between 180 and 450 HV), and only some heat-treated martensitic SS grades show high hardness (up to 700 HV) associated with their high carbon content (up to 1.2% wt) which can foster the formation of martensite^1^. Briefly, the high carbon content decreases the martensite transformation temperature, enabling a fully martensitic microstructure at high cooling rates and obtaining a wear-resistant microstructure. To further enhance the wear performance of the matrix, hard phases (such as carbides) can be incorporated into the steel matrix.

Implementing additive manufacturing (AM) allows the production of novel materials with desired compositions, microstructural features, and superior mechanical properties^5,6^. For instance, powder-bed fusion (PBF), one of the most commercialised AM processes, can deposit pre-alloyed powders to form a near-net-shape component by melting the powder using a heat source such as laser or electron beam^7^. Several studies have shown that AM-processed SS parts can be superior to those conventionally manufactured counterparts. For example, AM- processed austenitic SS has been shown to have improved mechanical properties due to the finer microstructure (i.e., Hall–Petch relation)^3,8,9^. The heat treatment in AM-processed ferritic SS promoted the formation of additional precipitates, providing mechanical properties similar to conventional counterparts^3,10^. AM-processed duplex SS having high strength and hardness have been introduced, where the improved mechanical properties are attributed to the Cr-rich intermetallic phases within the microstructure^11^. Further, improved mechanical properties for AM-processed martensitic SS and precipitation hardened SS can be obtained by controlling the retained austenite within the microstructure and optimising AM processing and heat treatment parameters^3,12–14^.

To date, the tribological performance of AM-processed austenitic SS has received much attention compared to that of other SS. The tribological behaviour of laser powder-bed fusion (L-PBF)-processed 316L was investigated as a function of AM processing parameters. It was shown that the minimisation of porosity by reducing the scanning speed or increasing the laser power both promoted wear resistance^15,16^. Li et al.^17^ performed dry sliding wear tests under different parameters (loads, frequencies, and temperatures) and showed that the primary wear mechanism was abrasion at room temperature, while the increase of sliding speed and temperature promoted oxidation. The developed oxide layer provided a bearing effect, and the friction forces were reduced with increasing temperature, while the wear rate was increased at higher temperatures. In other studies, the addition of TiC^18^, TiB_2_^19^ and SiC^20^ particles into the L-PBF-processed 316L matrix promoted the wear resistance due to the development of a compacted strain-hardened tribolayer with increasing volume ratio of hard particles. A protective oxide layer was also observed in L-PBF-processed precipitation hardened^12^ and duplex SS^11^, while it was shown that wear resistance could be enhanced by limiting the amount of retained austenite through post-heat treatment^12^. As summarised here, the literature has mainly focused on the tribological behaviour of the 316L SS family, while there is a paucity of data on the tribological behaviour of AM-processed martensitic SS family with much higher carbon content.

Electon beam melting (EBM), a technique similar to L-PBF, enables the formation microstructures with refractory carbides (such as vanadium- and chromium-rich carbides) since it is capable of achieving higher temperatures and scanning speeds than laser beam^21,22^. The available literature on EBM-processed SS is primarily concerned with identifying the optimal EBM processing paramaters for obtaining crack- and pore-free microstructures with improved mechanical properties^23–26^, whereas limited work is available for the tribological performance of EBM-processed SS. Thus far, the wear mechanisms of an EBM-processed, high carbon martensitic SS under limited conditions have been investigated, where it was reported that severe plastic deformation occurred with abrasion (sandpaper media testing), dry and slurry erosion conditions^27^.

In the present study, the wear and friction performance of the aftermentioned EBM-processed high carbon martensitic stainless steel has been examined under dry sliding conditions. First, the microstructural features were characterised using scanning electron microscopy (SEM), energy-dispersive X-ray spectroscopy (EDX), X-ray diffraction and image analysis. The data obtained from these methods were then used to form the basis of tribological behaviour observations made by conducting dry reciprocating tests at different loads, and finally, the worn surface morphologies were investigated using SEM–EDX and laser profilometry. The wear rate was quantified and compared to a similarly processed martensitic tool steel. This has been done to formulate a basis of comparison between this SS system, and that more commonly employed for wear resistance with the same type of processing history. Finally, cross-sectional hardness maps of the wear tracks were demonstrated using a hardness mapping algorithm, revealing the plastic deformation that occurred during the contact. It has to be noted that the tribological tests of this study are performed to provide a deeper understanding about the tribological behaviour of this novel material and not to simulate a particular application. The present study contributes to advance the current state of knowledge about the tribological behaviour of a novel AM-processed martensitic stainless steel designed specifically for wear-related applications that need to be operated under corrosive environments.

## Materials and method

### Materials and sample preparation

EBM-processed high carbon martensitic stainless steel (HCMSS) samples (commercially designated Vibenite^®^ 350) developed and supplied by VBN Components AB, Sweden. The nominal chemical composition of the samples is 1.9 C, 20.0 Cr, 1.0 Mo, 4.0 V, 73.1 Fe (wt%). First, dry sliding test specimens (40 mm × 20 mm × 5 mm) were produced from as-received rectangular coupons (42 mm × 22 mm × 7 mm) without any post heat treatment using electrical-discharge machining (EDM). Then, the specimens were ground sequentially with SiC abrasive paper grades ranging from 240 to 2400 P to obtain a surface roughness (Ra) of ~ 0.15 μm. In addition, EBM-processed high carbon martensitic tool steel (HCMTS) samples (commercially designated Vibenite^®^ 150) with a nominal chemical composition of 1.5 C, 4.0 Cr, 2.5 Mo, 2.5 W, 4.0 V, 85.5 Fe (wt%) were also prepared with the same methodology. HCMTS contains 8% volume of carbides and is only used to provide a comparison of the wear rate data of the HCMSS.

### Microstructural and mechanical characterisation

Microstructural characterisation of HCMSS was carried out using an SEM (FEI Quanta 250, USA) equipped with an energy dispersive X-ray (EDX), XMax80 detector by Oxford instruments. Three micrographs comprising 3500 μm^2^ were randomly taken in back-scattered electron (BSE) mode, and then area fraction (i.e. volume fraction), size and shape of the microstructural features (i.e. carbides) were analysed using image analysis (ImageJ^®^)^28^. It was assumed that the area fraction is equal to the volume fraction due to the morphology of features observed. Further, the shape factors of the carbides were calculated using the shape factor (Sh_fa_) equation:

$${Sh}_{fa}=4\pi \frac{{A}_{i}}{{P}_{i}^{2}}$$

here, A_i_ is the area of the carbide (μm^2^), and P_i_ is the perimeter of the carbide (μm)^29^. Powder X-ray diffraction (XRD) was performed to identify the phases using an x-ray diffractometer (Bruker D8 Discover with LynxEye 1D Strip detector) with Co-Kα radiation (λ = 1.79026 Å). The samples were scanned over a 2θ range from 35° to 130° with a step size of 0.02° and a step time of 2 s. The XRD data were analysed using Diffract.EVA software updated with a crystallographic database in 2021. In addition, a Vickers hardness tester (Struers Durascan 80, Austria) was used for micro-hardness testing. 30 indents with a spacing of 0.35 mm were conducted at 5 kgf for 10 s on the metallographically prepared specimens following the ASTM E384-17 standard^30^. The microstructural characterization of HCMTS has been described previously by the authors^31^.

### Tribological characterisation

A ball-on-plate tribometer (Bruker Universal Mechanical Tester Tribolab, USA) was employed to perform dry reciprocating wear tests, with a configuration detailed elsewhere^31^. The test parameters were as follows: 3 N load, 1 Hz frequency, 3 mm stroke for 1 h according to ASTM G133-05 standard^32^. Alumina balls (Al_2_O_3_ with precision grade 28/ISO 3290) with a diameter of 10 mm were used as counterbody and their macro-hardness was ~ 1500 HV and surface roughness (Ra) was ~ 0.05 μm, as supplied by Redhill Precision, Czech Republic. An alumina counterbody was selected to prevent the influence of oxidative effects that might originate from the counterbody and provide a better understanding of the wear mechanisms of the coupon under severe wear conditions. It should be noted that the test parameters are the same as in Ref.^8^ to compare the wear rate data with this existing study. Additionally, a set of reciprocating tests with an applied load of 10 N was performed to examine the tribological performance at higher loads, where the other test parameters were kept the same. The initial Hertzian contact pressures were 7.7 MPa and 11.5 MPa at 3 N and 10 N, respectively. During the wear tests, the friction forces were recorded at a frequency of 45 Hz, and the average coefficient of friction (CoF) values was calculated. Three measurements were performed for each load under ambient conditions.

The wear tracks were examined using the aforementioned SEM, while EDX analysis was performed to analyse the elemental composition of the worn surfaces using Aztec acquisition software. The worn surfaces of the counterbodies were examined with an optical microscope (Keyence VHX-5000, Japan). A non-contact laser profilometer (NanoFocus μScan, Germany) was used to scan the wear tracks with a vertical resolution of ± 0.1 μm in z and 5 μm in x and y directions. Surface profile maps of the wear tracks were generated in Matlab^®^ using the x, y, z coordinates obtained from the profilomentry measurements. Several perpendicular line profiles to the wear track extracted from the surface profile maps to calculate the wear volume loss of the wear tracks. The volume loss calculated as the product of the average cross-sectional area of the line profiles and the length of the wear track, further details of this methodology previously described by the authors^33^. From this, the specific wear rate (k) was obtained using the following formula:

$$k=\frac{V}{W\times L}$$

here, V is the wear volume loss (mm^3^), W is the applied load (N), L is the sliding distance (mm), and k is the specific wear rate (mm^3^/Nm)^34^. The friction data and surface profile maps for the HCMTS were included in the supplementary material (Supplementary Figs. S1 and Figs. S2), which were used to compare the wear rate of the HCMSS.

### Mechanical characterisation of wear affected zone

**Figure 1:**
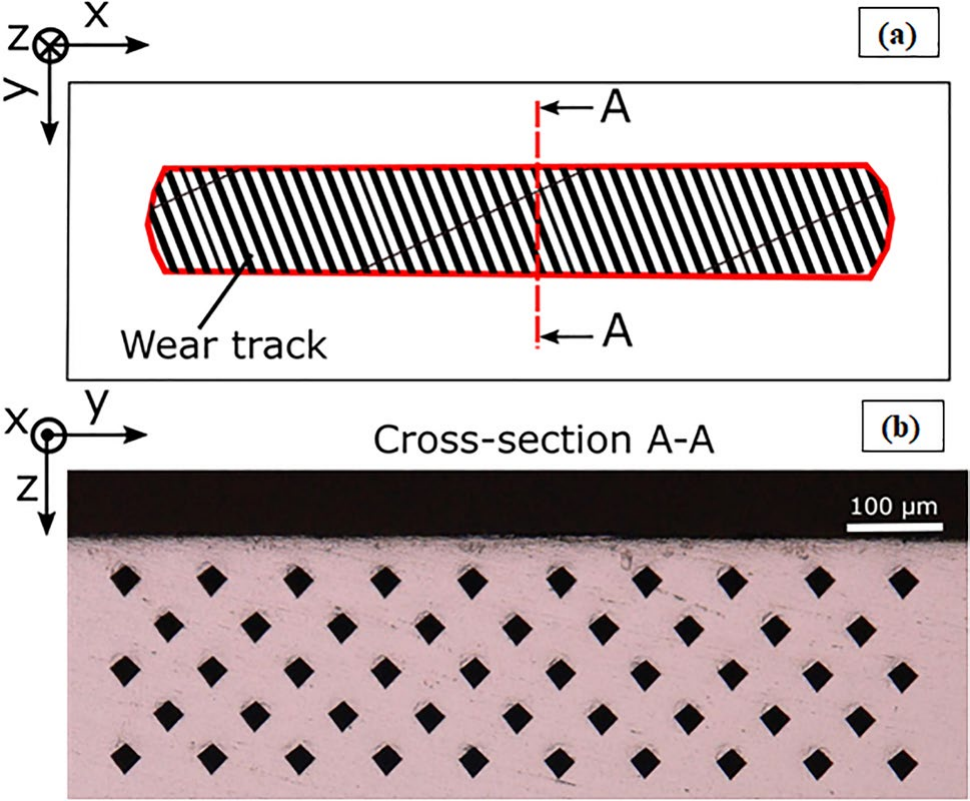
Schematic of wear track showing the position of the cross-section (**a**), an optical micrograph of the hardness mapping showing the footprint of the idents in the cross-section (**b**)**.**

In the present study, cross-sectional hardness maps of the wear tracks were used to demonstrate the plastic deformation behaviour of the wear affected zone (i.e., strain hardening due to the contact pressure). The worn specimens were cut with an aluminium oxide cutting wheel using a cut-off machine (Struers Accutom-5, Austria) and ground with SiC abrasive paper grades ranging from 240 to 4000 P over the thickness of the coupons. Microhardness measurements with 0.5 kgf for 10 s and spacing of 0.1 mm were performed following the ASTM E348-17 standard. The indents were located on a rectangular grid of 1.26 × 0.3 mm^2^ and ~ 60 um below the surface (Fig. 1), and then hardness maps were visualised using a customised Matlab^®^ code described elsewhere^35^. Further, the cross-sectional microstructures of the wear affected zone were examined using an SEM.

## Results and discussion

### Microstructural and mechanical properties

**Figure 2:**
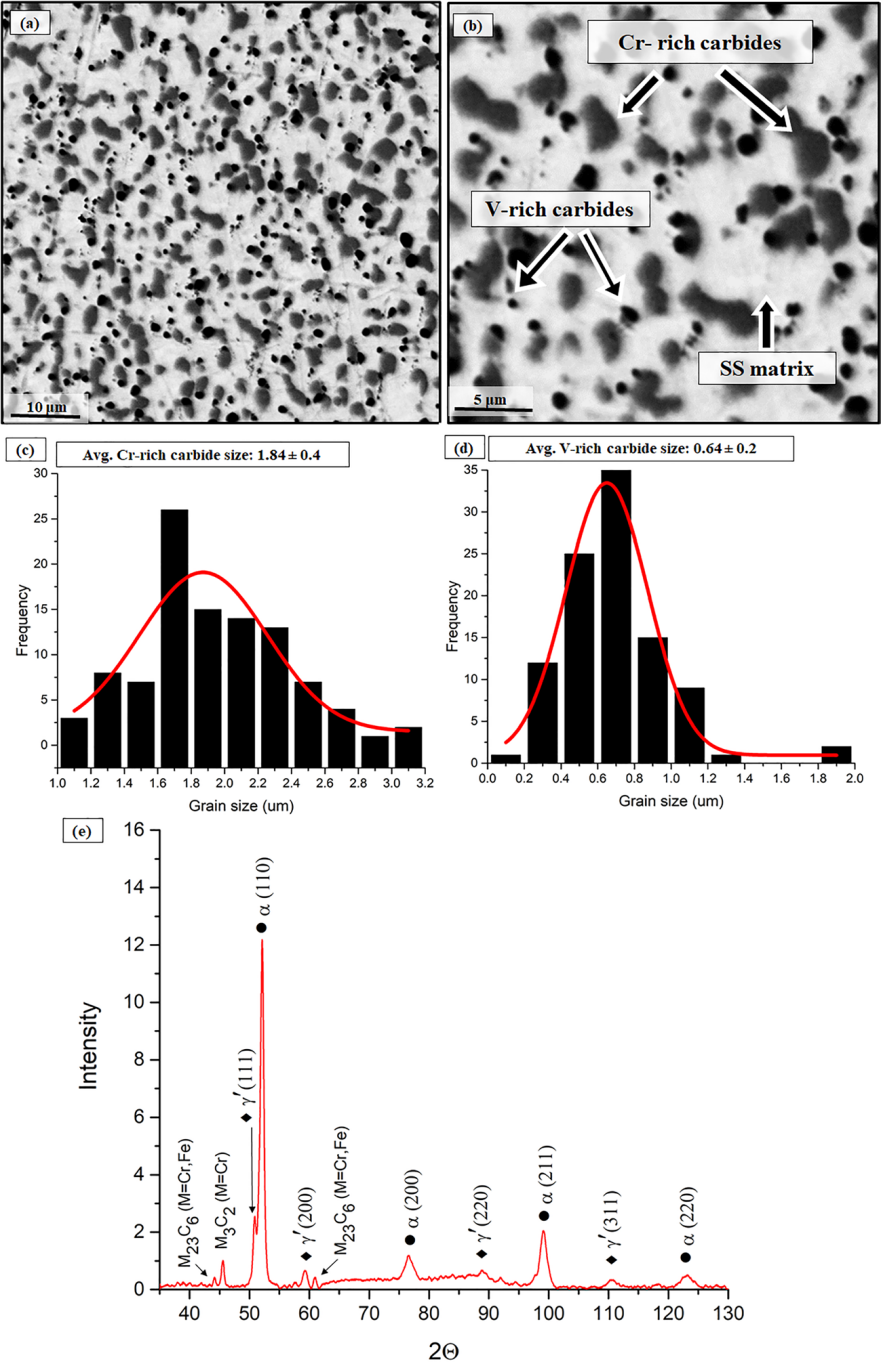
Microstructures of EBM-processed high carbon martensitic stainless steel (**a**) at low magnification and (**b**) at high magnification showing Cr-rich, V-rich carbides, and stainless-steel matrix (back-scattered electron mode). Histograms revealing grain size distribution of (**c**) Cr-rich and (**d**) V-rich carbides. XRD pattern showing the presence of martensite, retained austenite, and carbides within the microstructure (**d**).

The microstructure of the EBM-processed HCMSS consists of a homogenous network of carbides surrounded by a matrix (Fig. 2a,b). The EDX analysis shows that the grey coloured and dark coloured carbides are Cr-rich and V-rich carbides, respectively (Table 1). As calculated via image analysis, the volume fraction of carbides is estimated to be ~ 22.5% (~ 18.2% Cr-rich carbides and ~ 4.3% V-rich carbides). The average grain sizes with standard deviation are 0.64 ± 0.2 μm and 1.84 ± 0.4 μm for V-rich and Cr-rich carbides, respectively (Fig. 2c,d). The V-rich carbides tend to be more circular with a shape factor (± standard deviation) of ~ 0.88 ± 0.03 since a shape factor with a value close to 1 corresponds to a circular carbide. In contrast, the Cr-rich carbides are not entirely circular, having a shape factor of ~ 0.56 ± 0.01, possibly due to the agglomeration. Martensite (α, BCC) and retained austenite (γ′, FCC) diffraction peaks are detected in the XRD pattern of the HCMSS, as shown in Fig. 2e. Further, the XRD diffractogram shows the presence of secondary carbides. The Cr-rich carbides are identified as M_3_C_2_ and M_23_C_6_ type carbides. Diffraction peaks of VC carbides have been reported at ≈ 43° and 63° according to the literature^36–38^, it is assumed that the VC peaks have been masked by the M_23_C_6_ peaks of Cr-rich carbides (Fig. 2e).

**Table 1:** EDX analysis of the Cr-rich and V-rich carbides of the EBM-processed high carbon martensitic stainless steel.

Element	Cr-rich carbides (wt%)	V-rich carbides (wt%)	SS matrix (wt%)
Fe	23.5	6.1	75.6
C	11.9	10.1	5.8
Cr	50.9	16.1	15.4
V	12.2	66.4	2.1
Mo	1.5	1.3	1.1
Total	100	100	100

The average micro-hardness is 625.7 + 7.5 HV5, showing a relatively high hardness compared to non-heat treated conventional processed martensitic SS (450 HV)^1^. The nanoindentation hardness of the V-rich carbides and Cr-rich carbides has been reported ranging between 12 and 32.5 GPa^39^ and 13–22 GPa^40^, respectively. Thus, the high hardness of EBM-processed HCMSS is attributed to the high carbon content that promoted carbide network formation. In conclusion, the EBM-processed HCMSS presents promising microstructural characteristics and hardness without any additional post-heat treatment.

### Friction performance

**Figure 3:**
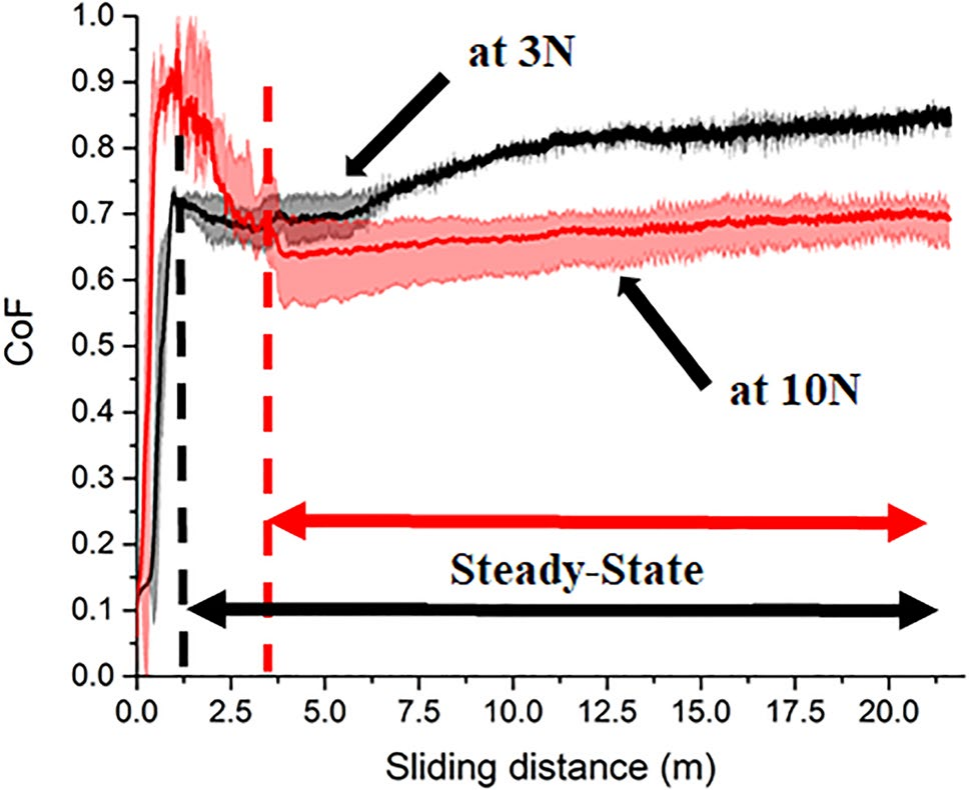
The coefficient of friction against the sliding distance of EBM-processed HCMSS samples at 3 N and 10 N; steady-state stages are annotated for each curve.

The mean coefficient of friction (CoF) curves of the samples at 3 N and 10 N are presented in Fig. 3; the semi-transparent shading indicates the range of the minimum and maximum friction values. Each curve demonstrates running-in and steady-state stages. The running-in stage ends at 1.2 m with a CoF (± standard deviation) of 0.41 ± 0.24 at 3 N, while it ends at 3.7 m with a CoF of 0.71 ± 0.16 at 10 N, and then the steady-state stage occurs where the friction does not change that rapidly. The friction forces rapidly increase in the running-in stages at both 3 N and 10 N due to the small contact area and the initial plastic deformation of the asperities^41^, where higher friction forces and an extended sliding distance occur at 10 N possibly due to the higher surface damage compared to that of 3 N. The CoF in the steady-state stage is 0.78 ± 0.05 and 0.67 ± 0.01 for 3 N and 10 N, respectively. The CoF is almost stable at 10 N, while it gradually increases at 3 N. In the limited literature, the CoF of L-PBF-processed SS against ceramic counterbodies at low applied loads has been reported ranging between 0.5 and 0.72^8,20,42^, agreeing well with the measured CoF values of this study. The decrease of CoF (around 14.1%) with increasing load in the steady-state could be attributed to the surface degradation that occurred at the interface between the worn surface and counterbody, which is further discussed through the surface analysis of the worn samples in the following sections*.*

### Wear performance

**Figure 4:**
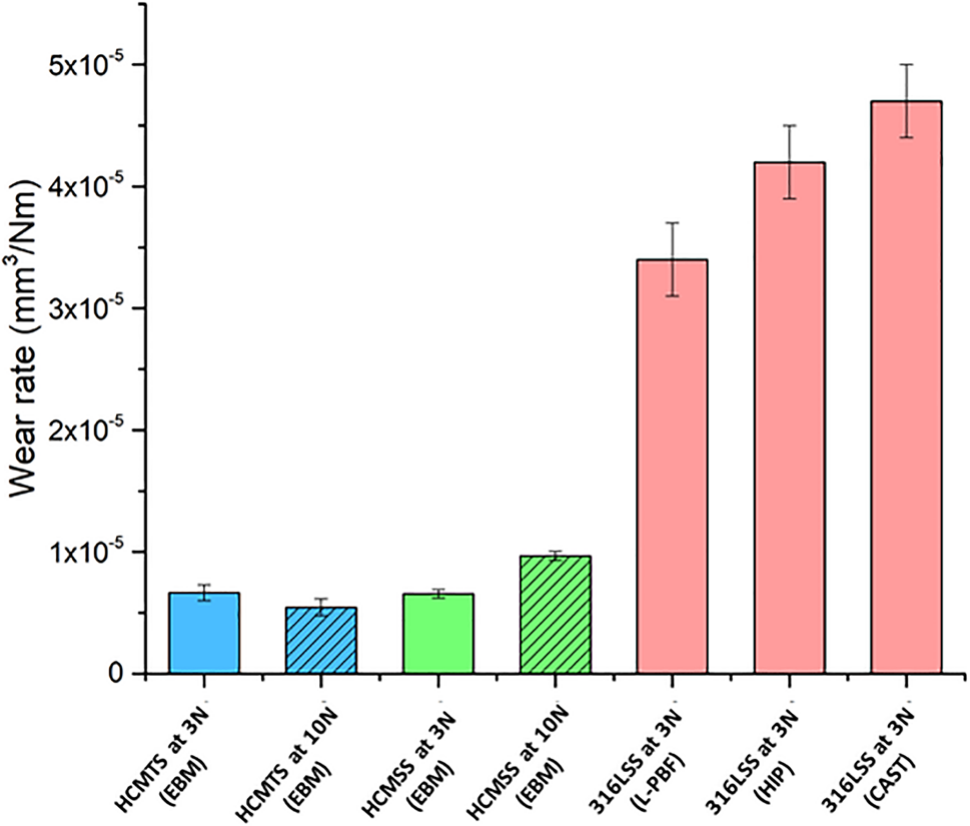
Specific wear rates of EBM-processed high carbon martensitic stainless steel (HCMSS), EBM-processed high carbon martensitic tool steel (HCMTS) and L-PBF-, cast- and high isotropic pressing (HIP)-processed austenitic stainless steel (316LSS) at different applied loads. The scatter bars show the standard deviation of the measured values. The data for austenitic stainless steel was retrieved from^8^.

The specific wear rate of HCMSS (625.7 HV) was estimated as 6.56 ± 0.33 × 10^–6^ mm^3^/Nm and 9.66 ± 0.37 × 10^–6^ mm^3^/Nm at 3 N and 10 N, respectively (Fig. 4). Thus, the wear rate increased with increasing load, agreeing well with the existing studies on L-PBF-processed austenitic and PH SS^17,43^. The wear rate at 3 N is lower around one fifth of the value of an L-PBF-processed austenitic SS (k = 3.50 ± 0.3 × 10^−5^ mm^3^/Nm, 229 HV) under the same tribological conditions, as reported in a previous study^8^. Furthermore, the wear rate of HCMSS at 3 N is significantly lower than conventional processed austenitic SS; more specifically, it is lower around one sixth and one seventh of the value of a high isotropic pressing—(k = 4.20 ± 0.3 × 10^−5^ mm^3^/Nm, 176 HV) and a cast—(k = 4.70 ± 0.3 × 10^−5^ mm^3^/Nm, 156 HV) processed austenitic SS, respectively^8^. The improved wear-resistance of HCMSS compared to those studies in the literature is attributed to high carbon content and formed carbide network, resulting in a higher hardness than those AM-processed and conventionally processed austenitic SS. To further examine the wear rate of the HCMSS samples, similarly processed high carbon martensitic tool steel (HCMTS) samples (with a hardness of 790 HV) were tested under similar conditions (at 3 N and 10 N) for comparison; surface profile maps of HCMTS included in the supplementary material (Supplementary Fig. S2). The wear rate of HCMSS (k = 6.56 ± 0.34 × 10^–6^ mm^3^/Nm) was almost the same compared to the wear rate of HCMTS at 3 N (k = 6.65 ± 0.68 × 10^–6^ mm^3^/Nm), indicating an exceptional wear resistance. This performance was primarily attributed to the microstructural features of HCMSS (i.e. a high carbide content, the size, shape and distribution of carbide particles within the matrix as described in Sect. 3.1). As previously reported^31,44^, the carbide content influences the width and depth of the wear track as well as the micro-abrasive wear mechanisms. However, the carbide content was insufficient to protect the matrix at 10 N, resulting an increase in the wear rate. In the section that follows, worn surface morphologies and topographiesare used to explain the dominant wear and deformation mechanisms affecting the wear rate of the HCMSS. The wear rate of HCMSS (k = 9.66 ± 0.37 × 10^–6^ mm^3^/Nm) was higher compared to the the wear rate of the HCMTS (k = 5.45 ± 0.69 × 10^–6^ mm^3^/Nm) at 10 N. Comparatively, these wear rates are still quite high: chromium-based and Stellite coatings exhibit lower wear rates than the HCMSS under similar testing conditions^45,46^. Finally, the wear rate of the counterbody was negligible due to the high hardness of alumina (1500 HV), and there were signs of material transfer from the sample to the alumina balls.

Despite the fact that hardface coatings, such as chromium-based and Stellite coatings, can provide higher wear-resistance than AM-processed alloy systems, AM enables (1) microstructural refinement, particularly with alloys having a constituent with big differences in density, (2) the reduction of subtractive operations on a final part, and (3) the production of novel surface topologies, such as built-in hydrodynamic bearings. Moreover, AM offers geometric design flexibility. This study is particularly novel and significant as it is critical to reveal the wear behaviour of these newly developed metal alloys via EBM, where the current literature is very limited.

### Wear mechanisms

**Figure 5:**
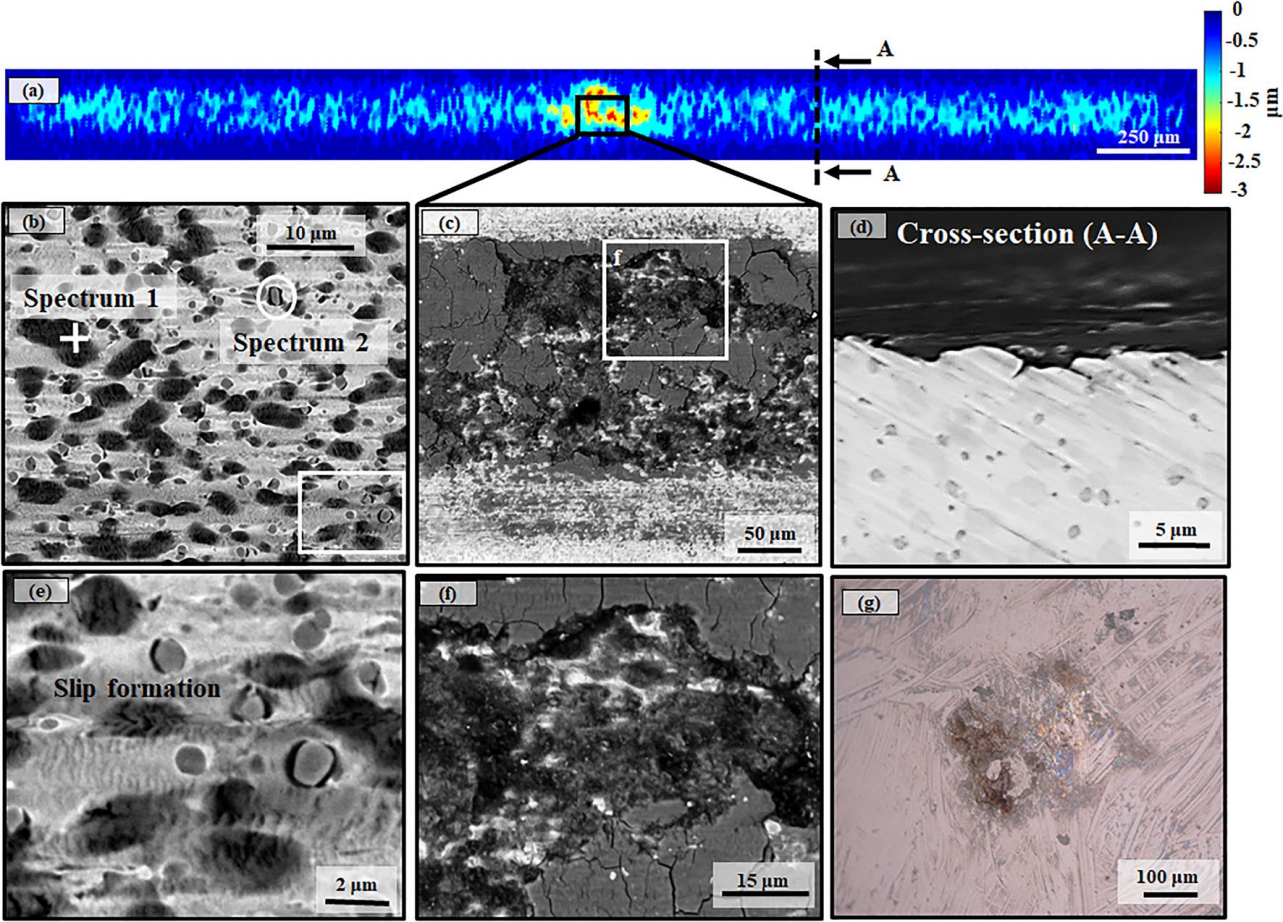
Surface profile map (**a**) and micrographs of worn surface morphologies (**b**–**f**), the cross-section of wear track (**d**) in BSE mode for EBM-processed high carbon martensitic stainless steel at 3 N and the optical microscopy of worn surface of the alumina ball at 3 N (**g**).

The worn surface morphologies and topography of the worn samples at 3 N are shown in Fig. 5, where the dominant wear mechanism was abrasion followed by oxidation. First, the steel matrix was plastically deformed, and then the steel matrix was removed, causing grooves with a depth ranging between ~ 1 and 3 μm as shown in the surface profile map (Fig. 5a). The removed material remained at the interface of the tribosystem, forming a tribolayer consisting of small Fe-rich oxide islands around Cr-rich and V-rich carbides (Fig. 5b and Table 2) due to the friction heat from the continuous sliding, as also reported for L-PBF-processed austenitic SS^15,17^. Figure 5c indicates the intense oxidation that occurred in the centre of the wear track. Thus, either the material removal was accelerated due to the fracturing of the tribolayer (i.e., oxide layer) (Fig. 5f) or the material removal progressed in the weak regions within the microstructure, promoting the formation of the tribolayer. In both cases, the fracture of the tribolayer generated wear debris at the interface, which may be the reason for the increasing trend of the CoF in the steady-state at 3 N (Fig. 3). Further, there were signs of three-body abrasion caused by the oxide and loose wear particles on the wear track, eventually forming micro-scratches on the matrix (Fig. 5b,e)^9,12,47^.

**Table 2:** EDX analysis of corresponding spectra shown in the worn surface of EBM-processed high carbon martensitic stainless steel at 3 N.

Element	Spectrum 1 (wt%)	Spectrum 2 (wt%)
Fe	40.4	7.1
O	24.6	12.1
V	5.1	59.8
Cr	22.3	10.7
Mo	1.0	1.1
C	6.6	9.2
Total	100.0	100.0

Slip bands were formed on the steel matrix, indicating the plastic deformation due to wear (Fig. 5e). Similar results were also reported in a study on the wear behaviour of L-PBF-processed austenitic SS^47^. The re-orientation of the V-rich carbides also indicated the plastic deformation of the steel matrix during the sliding (Fig. 5e). The cross-section micrograph of the wear track revealed the existence of minor circular pits surrounded by micro-cracks (Fig. 5d), possibly due to the excessive plastic deformation of the near-surface. There was limited material transfer to the alumina ball, while the ball remained undamaged (Fig. 5g).

**Figure 6:**
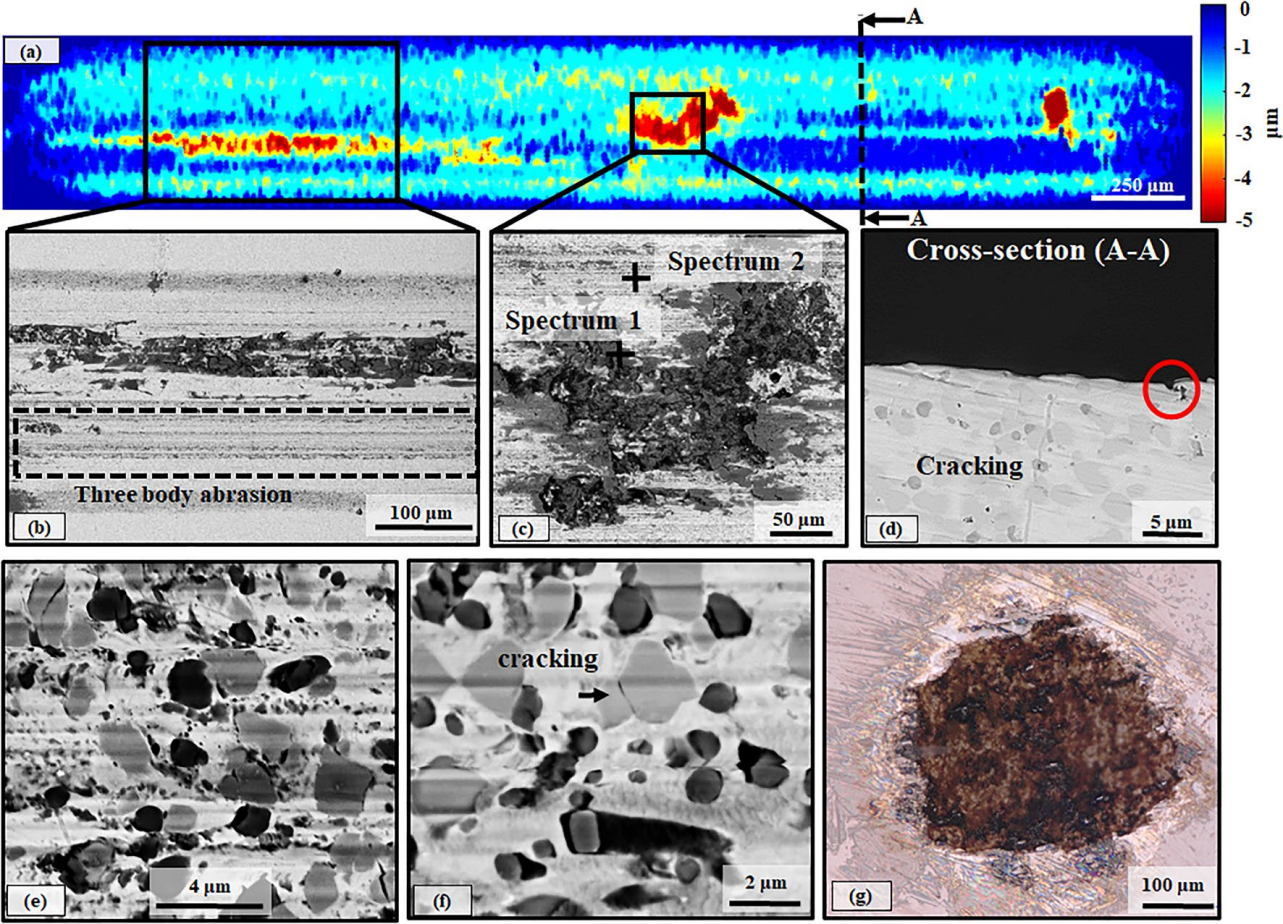
Surface profile map (**a**) and micrographs of worn surface morphologies (**b**–**f**), the cross-section of wear track (**d**) in BSE mode for EBM-processed high carbon martensitic stainless steel at 10 N and the optical microscopy of the worn surface of the alumina ball at 10 N (**g**).

The wear width and depth of the samples increased with increasing load (at 10 N), as shown in the surface topography map (Fig. 6a). Abrasion and oxidation were still the dominant wear mechanisms, while the increased number of micro-scratches on the wear track suggests that three-body abrasion was also significant at 10 N (Fig. 6b). The EDX analysis showed the formation of Fe-rich oxide islands. The Al peaks in the spectrum confirmed that material transfer occurred from the counterbody onto the sample (Fig. 6c and Table 3) at 10 N, which was not observed at 3 N (Table 2). Three-body abrasion was caused by wear debris particles from the oxide islands and the counterbody, where detailed EDX analysis revealed material transfer from the counterbody (Supplementary Figure S3 and Table S1). The development of the oxide islands was associated with high depth pits, as also observed at 3 N (Fig. 5). Carbide cracking and fragmentation occurred mainly for Cr-rich carbides at 10 N (Fig. 6e,f). Furthermore, V-rich carbides detached and abraded the surrounding matrix and then further caused three-body abrasion. In the cross-section of the track (Fig. 6d), there was also a pit (highlighted with a red circle) with a similar size and shape with the size of V-rich carbides (please see carbide size and shape analysis in *Sect. 3.1*), showing that V-rich carbides were potentially dislodged from the matrix at 10 N. The circular shape of V-rich carbides promoted the pull-out effect, while the agglomerated Cr-rich carbides were susceptible to cracking (Fig. 6e,f). This fracturing behaviour suggested that the matrix's capacity to withstand plastic deformation was already exceeded, and the microstructure did not provide adequate toughness at 10 N.Vertical cracking in the subsurface (Fig. 6d) indicated the intensity of the plastic deformation that occurred during the sliding. Some material was transferred from the wear track onto the alumina ball with increasing load (Fig. 6g), which may be the underlying reason for the decreased CoF values at 10 N in the steady-state (Fig. 3).

**Table 3:** EDX analysis of corresponding spectra shown in the worn EBM-processed high carbon martensitic stainless steel at 10 N.

Element	Spectrum 1 (wt%)	Spectrum 2 (wt%)
Fe	41.1	77.1
O	23.6	1.3
V	2.2	1.7
Cr	11.7	14.1
Mo	0.7	0.9
C	15.7	4.8
Al	5.0	–
Total	100.0	100.0

### Mechanical properties of wear affected zone

During sliding wear, the surface is subjected to compressive and shear stresses induced by the counterbody, resulting in significant plastic deformation beneath the worn surface^34,48,49^. Consequently, strain-hardening within the subsurface can occur due to the plastic deformation, influencing wear and deformation mechanisms that govern the wear behaviour of materials. Thus, in the present study, cross-sectional hardness mapping (as detailed in *Sect. 2.4*) was performed to identify the development of a plastically deformed zone (PDZ) beneath the wear track as a function of load. Since, clear signs of plastic deformation below the wear track were observed (Figs. 5d, 6d), specifically at 10 N, as discussed in the previous sections.

**Figure 7:**
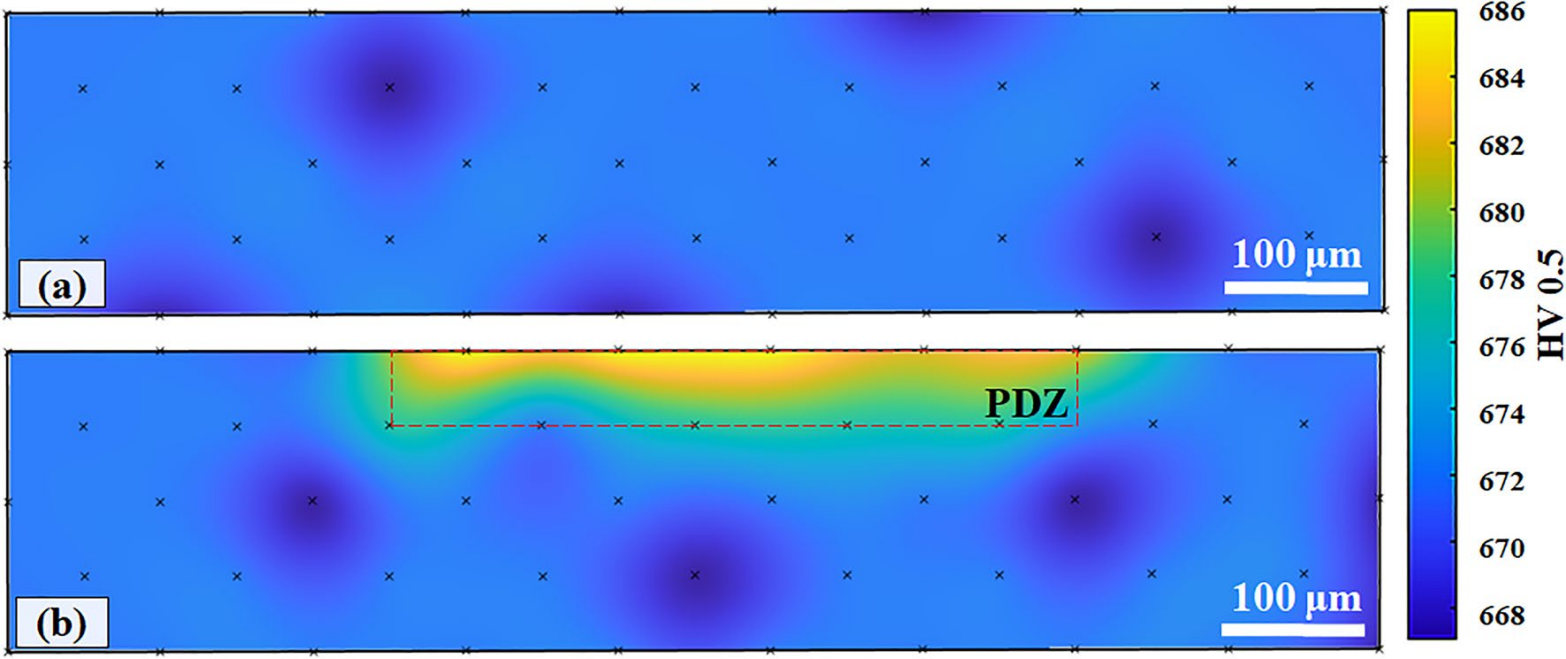
Cross-sectional hardness maps of the wear track of EBM-processed high carbon martensitic stainless steel at 3 N (**a**) and 10 N (**b**).

In Fig. 7, the cross-sectional hardness maps of the wear track of EBM-processed HCMSS at 3 N and 10 N are given. It is noteworthy to state that these hardness values are used as an indicator to evaluate the strain-strengthening effect. The hardness variation beneath the wear track was between 667 and 672 HV at 3 N (Fig. 7a), indicating that strain hardening was insignificant. Presumably, the applied hardness measurement method was not able to detect any hardness change due to the low resolution (i.e. spacing between the idents) of micro-hardness mapping. By contrast, a PDZ zone having hardness values between 677 and 686 HV and the maximum depth of 118 μm and length of 488 μm was observed at 10 N (Fig. 7b), correlating well with the width of the wear track (Fig. 6a). Similar findings on the variation of the size of PDZ as a function of load was reported in a study on the wear behaviour of L-PBF-processed SS^47^. It was shown that the presence of retained austenite played a role in the plasticity of AM-processed SS^3,12,50^ and that the retained austenite transformed to martensite under plastic deformation (transformation induced plasticity effect), enhancing the strain-hardening of steels^51^. As the HCMSS samples contain retained austenite according to the previously discussed XRD pattern (Fig. 2e), it is assumed that the retained austenite within the microstructure may have transformed to martensite during the contact, increasing the hardness in the PDZ (Fig. 7b). Further, the slip formation that occurred on the wear track (Figs. 5e, 6f) also indicates the plastic deformation by dislocation slipping under shear stresses was caused during the sliding contact. However, the shear stress generated at 3 N was insufficient to obtain a high dislocation density or transform the retained austenite to martensite on a scale observable by the methods employed; thus, the strain hardening is only observed at 10 N (Fig. 7b).

## Conclusions

The present study reveals the wear behaviour and the microstructural features of a novel EBM-processed high carbon martensitic stainless steel. Dry sliding wear tests were performed at different loads, and the worn samples were examined via electron microscopy, laser profilometry and cross-sectional hardness mapping of the wear tracks.

The microstructural analysis showed a homogeneous distribution of Cr-rich (~ 18.2% carbides) and V-rich (~ 4.3% carbides) carbides within the martensitic and retained austenite matrix and a relatively high micro-hardness. The dominant wear mechanisms were abrasion and oxidation at low applied load, while three-body abrasion induced by pulled-out V-rich carbides and loose particle oxides also contributed to the wear with increasing load. The wear rate was superior to L-PBF-processed and conventional-processed austenitic SS; it was even similar to the EBM-processed tool steel at low applied load. The CoF values were decreased with load increase due to material transfer over the counterbody. A plastic deformed zone was revealed beneath the wear track through a cross-sectional hardness mapping approach. The possible grain refinement and phase transformation of the matrix could be further investigated using electron backscatter diffraction to better understand the strain-hardening effect. The low resolution of the micro-hardness mapping prevented the visualization of hardness in the wear affected zone at low applied load, and thus nano-indentation testing could provide the variation of hardness with higher resolutions by using the same approach.

For the first time, this study revealed a comprehisive analysis about the wear and friction performance of the novel EBM-processed high carbon martensitic stainless steel. Considering the geometric design freedom of AM and the potential to reduce the processing steps using AM, the present study can pave the way for the production and use of this type of novel materials in wear related applications ranging from shafts to plastic injection moulds with complex cooling channels.

## Supplementary Information

Supplementary Information.

## Data Availability

The dataset supporting this article can be found online at : https://doi.org/10.5281/zenodo.5767383.
