# Supplementary material for: Wear resistance of an additively manufactured high-carbon martensitic stainless steel
Source: Sci Rep. 2022 Jul 22;12:12554. doi: 10.1038/s41598-022-15621-9 (PMC9307504; doi:10.1038/s41598-022-15621-9)
Supplement: Supplementary file 1 — Supplementary Information. [file 41598_2022_15621_MOESM1_ESM.docx]

**Supplementary materials for**

**Wear resistance of an additively manufactured high-carbon martensitic stainless steel**

Eleftherios Iakovakis ^a, b*^, Egemen Avcu ^c, d, e*^, Matthew J. Roy ^a, e*^, Mark Gee ^b^, Allan Matthews ^d, e^

^a^ Department of Mechanical, Aerospace and Civil Engineering, The University of Manchester, Manchester, M13 9PL, UK

^b^ Department of Engineering, National Physical Laboratory, Teddington, TW11 0LW, UK

^c^ Department of Mechanical Engineering, Kocaeli University, Kocaeli, 41001, Turkey

^d^ Department of Materials, The University of Manchester, Manchester, M13 9PL, UK

^e^ Henry Royce Institute, Department of Materials, The University of Manchester, Manchester, M13 9PL, UK

**Supplementary Figures**


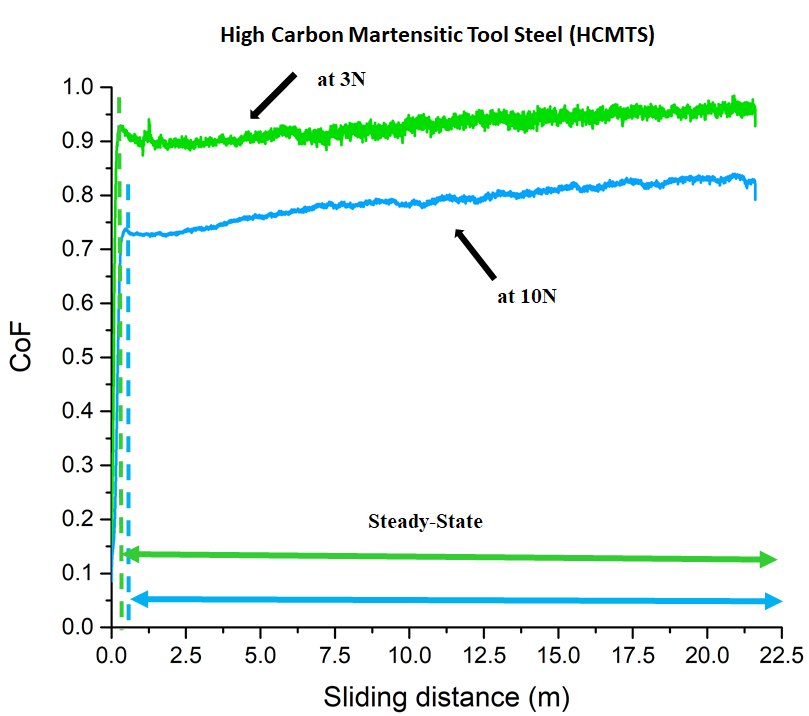


Figure S1. The coefficient of friction against the sliding distance EBM-processed high carbon martensitic tool steel samples at 3N and 10N; steady-state stages are annotated for each curve.


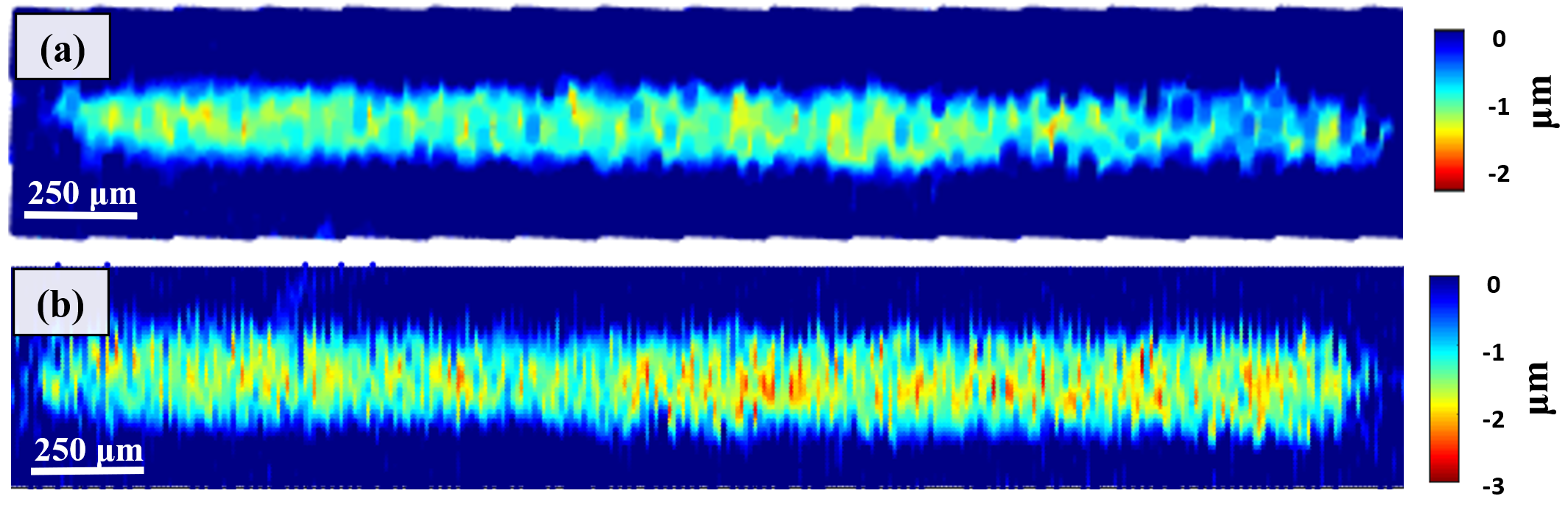


Figure S2. Surface profile maps for EBM-processed high carbon martensitic tool steel at 3N (a) and 10 N (b).


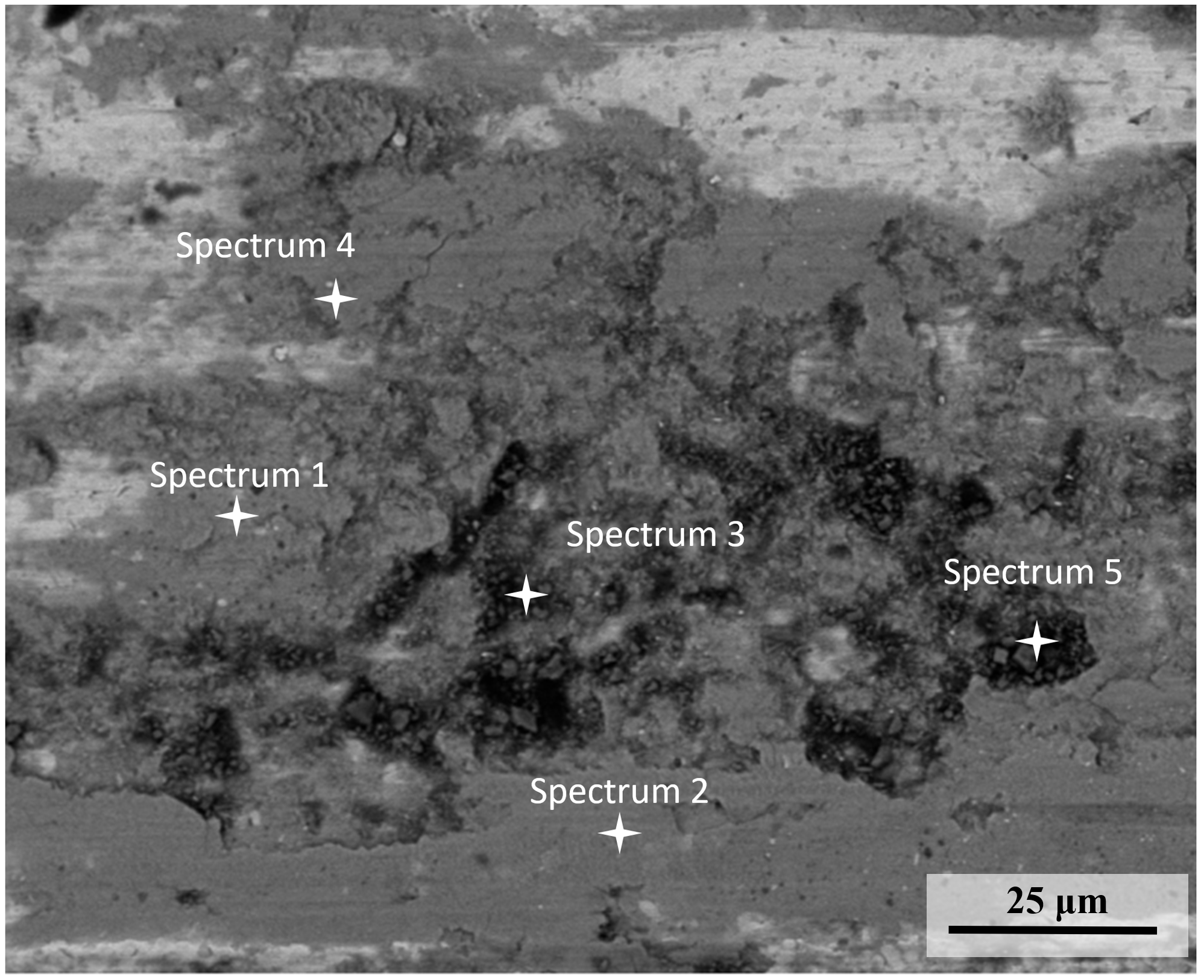


Figure S3: SEM micrographs of worn surface morphology of wear track in BSE mode for EBM-processed high carbon martensitic stainless steel at 10N, showing several wear debris particles and oxides; EDX spectrums are market on the micrographs.

**Supplementary Table**

Table S1: EDX analysis of corresponding spectrums shown in the worn surface of EBM-processed high carbon martensitic stainless steel at 10N (Figure S3).

| **Element** | **Spectrum 1 (wt. %)** | | **Spectrum 2 (wt. %)** | **Spectrum 3 (wt. %)** | **Spectrum 4 (wt. %)** | **Spectrum 5 (wt. %)** |
| --- | --- | --- | --- | --- | --- | --- |
| Fe | 47.4 | 44.2 | | 55.4 | 44.4 | 19.9 |
| O | 25.3 | 29.6 | | 14.8 | 27.0 | 34.0 |
| V | 2.9 | 3.6 | | 2.7 | 3.1 | 2.4 |
| Cr | 17.0 | 17.5 | | 17.9 | 17.0 | 15.7 |
| Mo | 1.1 | 1.0 | | 1.2 | 0.9 | 1.0 |
| Al | 5.7 | 3.3 | | 3.2 | 6.9 | 11.0 |
| Si | 0.6 | 0.5 | | 1.5 | 0.7 | 0.8 |
| S | - | 0.3 | | - | - | - |
| Ca | - | - | | 3.3 | - | 15.2 |
| Total | 100.0 | 100.0 | | 100 | 100 | 100 |
